# Supplementary material for: Identifying opportunity, capability and motivation of Sri Lankan 5th grade schoolteachers to implement in-classroom physical activity breaks: A qualitative study
Source: PLoS One. 2023 Jul 20;18(7):e0288916. doi: 10.1371/journal.pone.0288916 (PMC10359008; doi:10.1371/journal.pone.0288916)
Supplement: S2 File — (DOCX) [file pone.0288916.s002.docx]

## S 2: the Interview Guide (English Version)

| **Introduction**  **Opening Statement** | Thank you very much for informing me of your consent to participate in this interview. I would like to ask some questions about the teaching activities that you use or the activities that can be used to improve the academic performance, cognition skills and health-related outcomes of the scholarship exam level students. Any opinion that you would share is highly appreciated, as that will be helpful for our research team to develop an activity-based intervention for school kids. To make sure that I do not miss the data that you provide, would you like me to record our conversation? Of course, this conversation is confidential and, I ensure your privacy. Furthermore, I am using your responses only for the research-related purposes. I will permanently delete all the primary data that you have provided within one year, after completing this study. If you agree, may I ask you to verbally mention that you agree with the interview setting that I have explained to you now? Please feel free to ask any question if you have any at this stage. | |
| --- | --- | --- |
| **Transitioning to...** | **Question(s)** | **Probe(s)** |
| **Warm-up**  Socio-demographic information | First of all, would you like to share something about yourself with me? Including any information about your residency, teaching experience, education level, your age and any achievement of your teaching life... | How about your place of residence?  How many years have you been in teaching now?  Any special experiences that you want to share as a teacher? |
|  | Thank you very much for sharing your information with me. | |
| Approaching towards the interview topic with a convenient talk-related to academic performance | So, please tell me in general, what are expected to teach, how do you teach and how do you evaluate Grade Five students? | Are you always following the teachers’ guide, or do you use your own techniques in addition to the guidelines? |
| **Main body (Topic I to IV)** | Now, I would like to concentrate more on your current teaching practices specifically focusing on mathematics and reading. But, if you like to talk about any other subject in addition to math’s and reading, you are always welcome to speak 😊. | |
| **Topic I**  Academic Outcomes  (Mathematics and Reading) | 1. Please tell me, what are you expected to teach during the mathematics period? Is it just arithmetic or arithmetic and mathematics? | Can you explain to me more on that may be with an example? |
|  | 1. Can you describe the teaching methods that you use to teach mathematics? | Are those methods, formed by you or were you directed to do so by the teacher guides? |
|  | 1. How do you evaluate those students’ mathematics performance? | Do you use standardised tests recommended by some authority? Or do you use your own methods? |
|  | 1. Do you think that the students are needed to be improved in mathematics, if ‘yes’, in what ways? |  |
|  | Let’s talk about the reading performance of the students now. | |
|  | 1. What do you teach your students to improve their reading skills? | For example? |
|  | 1. How do you teach them the reading skills? | Are those methods, formed by you or were you directed to do so by the teacher guides? |
|  | 1. How do you evaluate the reading achievements of the students? | Do you use standardised tests recommended by some authority? Or do you use your own methods? |
|  | 1. Based on your evaluations did you find any need for improvement in reading? | That can be something related to students, or the teaching methods, or the curriculum. Can you tell me more now? |
| **Topic II**  Movement Behaviours and Health Outcomes  (Physical activity levels, Physical fitness/ Aerobic fitness, Stress/ test anxiety) | 1. I would like to bring your attention to the health of students. May I know, how would you like to define ‘health behaviour’ and ‘health’ of a student? |  |
|  | That’s a great definition. Let’s say health behaviour is all about engaging in PA, reducing sedentary behaviour. And health is all about improving physical fitness/ aerobic fitness, living with less exam-related anxiety, and stress. | |
|  | 1. Based on what I defined, could you please tell me whether you are teaching or motivating students to engage in physical activity, and improve physical fitness. 2. could you please tell me whether you are teaching or motivating students to manage stress or cope with test-anxiety. | Can you give me some examples?  Are you required to teach things related to physical activity levels and physical fitness?  What do you do to make them physically active and fit? |
|  | 1. How are you engaging or motivating them to improve mental health-enhancing activities such as coping with test anxiety or stress? | In what ways will you help them to manage stress or cope with test-anxiety?  How about building their confidence to be successful in accomplishing their activities? |
|  | 1. How would you know that they have a good physical and mental health? | How do you evaluate their physical health?  How do you evaluate their mental health?  Is that based on a curriculum guidance or is that based on your own decision-making capacity? |
|  | 1. I would like to get deeper into the mental health aspect a little bit. Do you think that the students are suffering from mental-health problems because of the Scholarship examination? | Can you tell me why do you think so?  May you share some experiences on those aspects with me? |
| **Topic III**  Perceptions on implementing IcPAB | 1. I would like to mention some activities that you can do in the classroom to help you improve the academic performance (more especially reading and mathematics) of the students. I will verbally demonstrate to you an activity now. What is your opinion on that activity? 2. Would you like to share some advice on how and in what ways I should plan those activities? | |
|  | 1. Should you have an opportunity to teach students using physical activities inside the classroom (which would last from five to ten minutes), are you willing to do so? | Can you please, tell me why?  What affected that decision of yours? |
|  | 1. Do you see any challenges in carrying out such activities? And why? |  |
|  | 1. What kind of solutions that we can think of to overcome such challenges? |  |
| **Cool-off** | 1. All right. Thank you very much for sharing your expertise with me. In addition to the questions that I asked, do you have anything else to share with me on this subject? | May be some other concerns of the academic performance, health of students or the teaching methods? |
| **Closure** | This is the end of our interview, then. I will be sent you a top-up fee to your mobile phone number as an appreciation for committing your time during this difficult time. Thank you very much. | |
