# Supplementary material for: Identifying opportunity, capability and motivation of Sri Lankan 5th grade schoolteachers to implement in-classroom physical activity breaks: A qualitative study
Source: PLoS One. 2023 Jul 20;18(7):e0288916. doi: 10.1371/journal.pone.0288916 (PMC10359008; doi:10.1371/journal.pone.0288916)
Supplement: S3 File — (DOCX) [file pone.0288916.s003.docx]

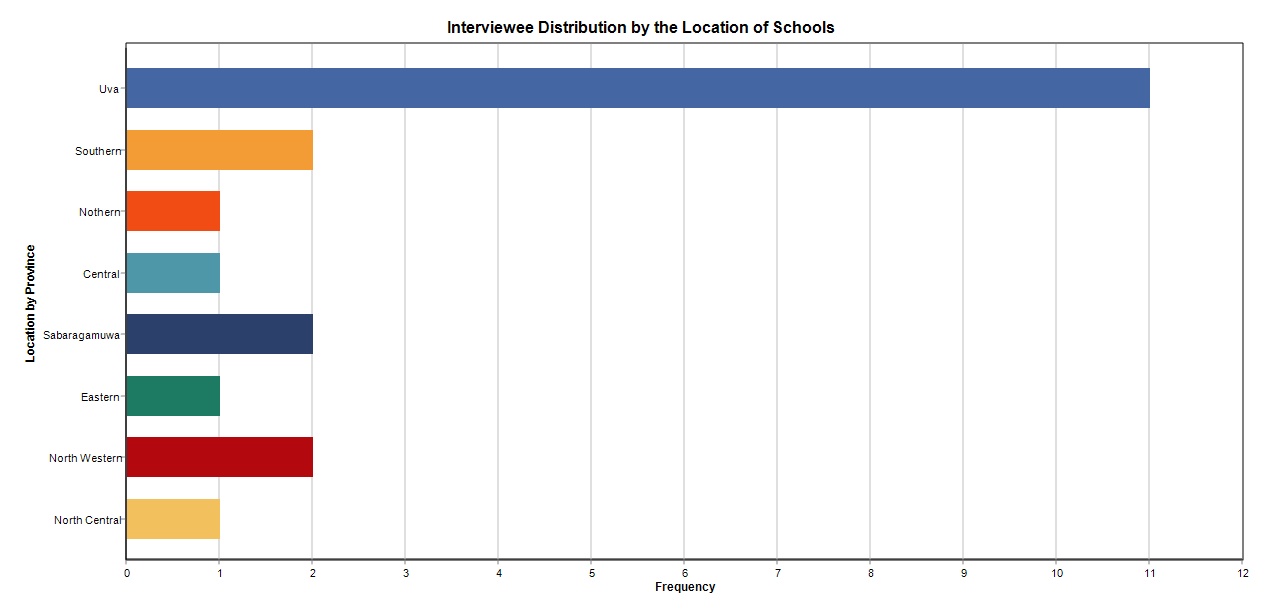


Fig 1: Residential representation of the respondents by province


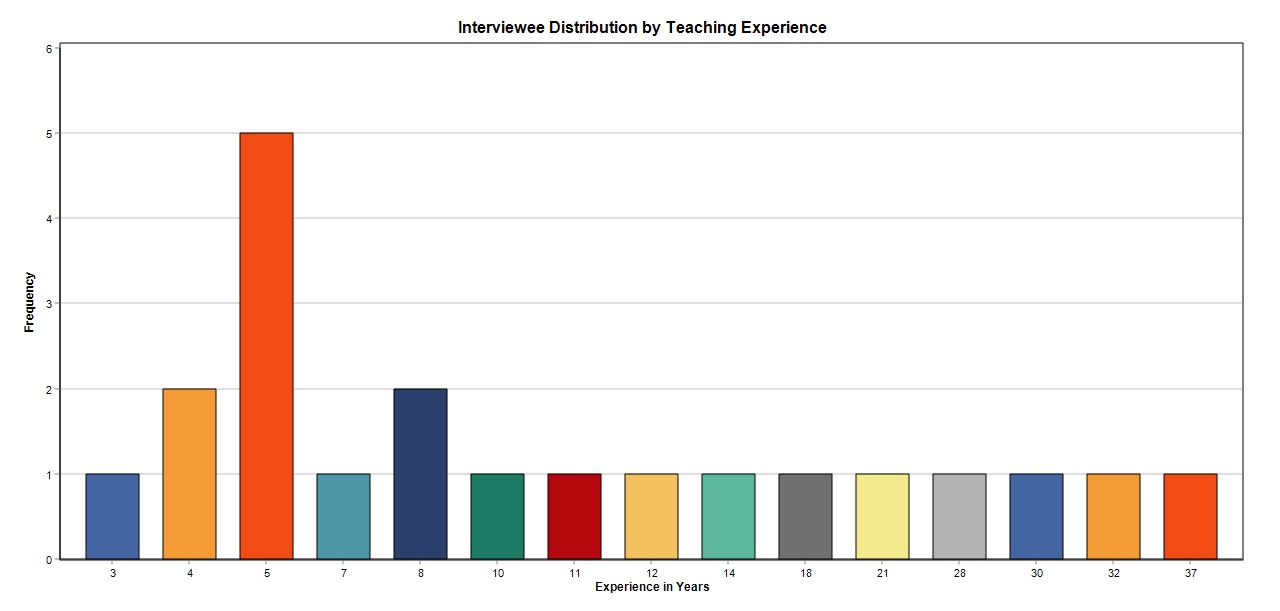


Fig 2: Teaching experience of the respondents in years


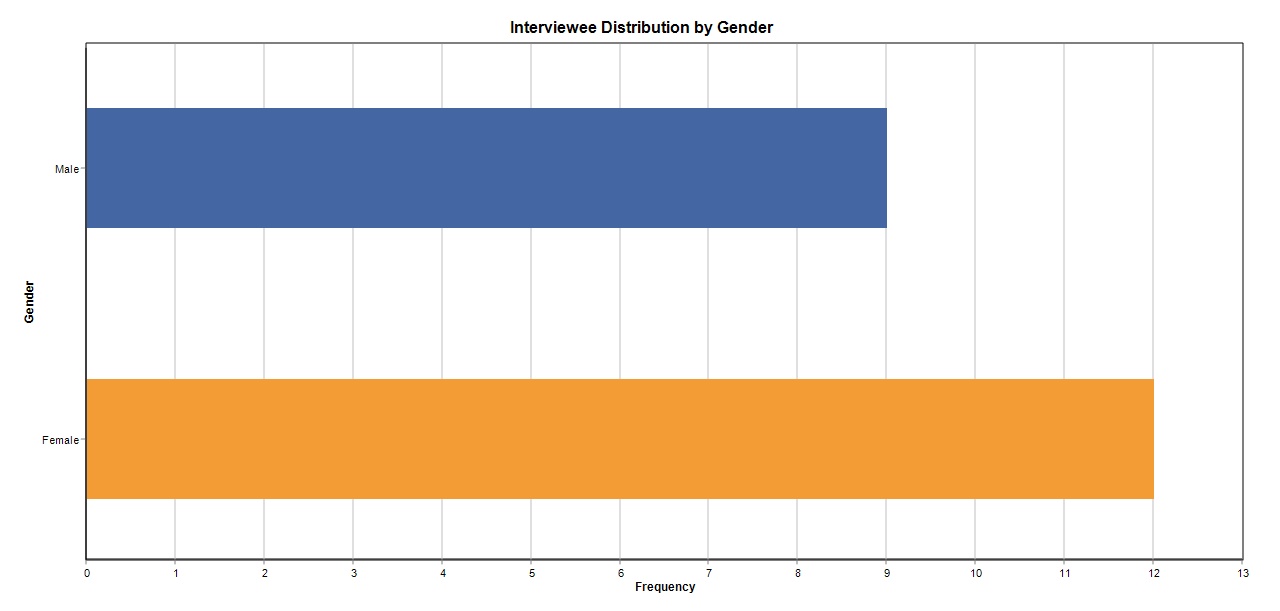


Fig 3: Gender of the respondents


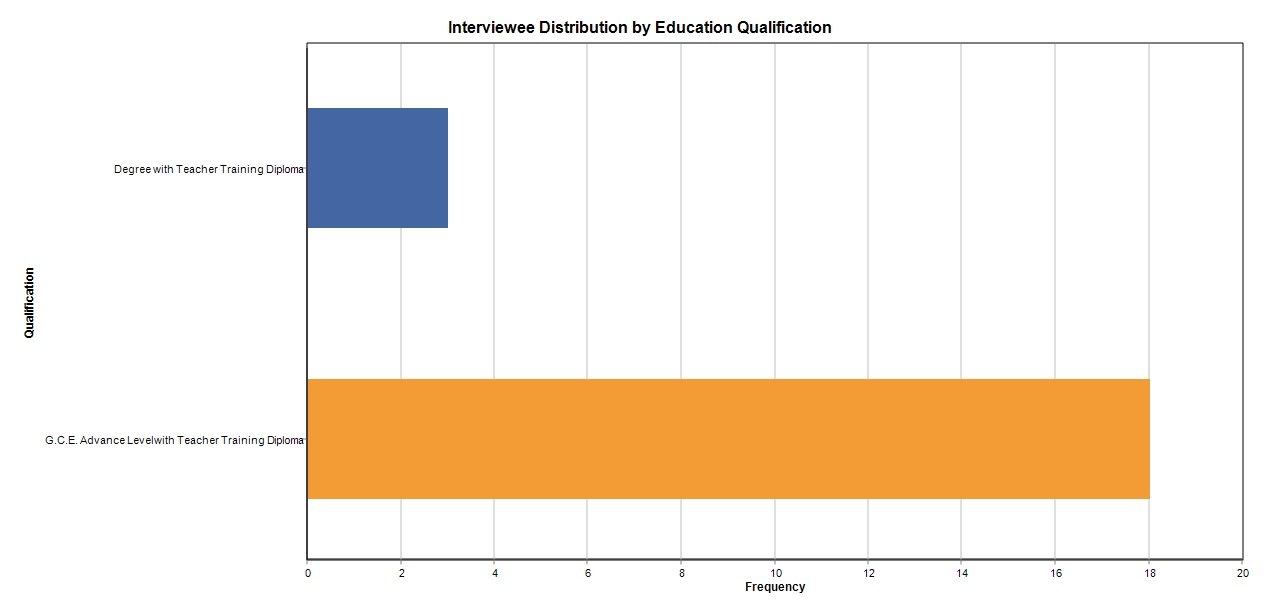


Fig 4: Highest education qualification of the respondents
