# Supplementary material for: Identifying opportunity, capability and motivation of Sri Lankan 5th grade schoolteachers to implement in-classroom physical activity breaks: A qualitative study
Source: PLoS One. 2023 Jul 20;18(7):e0288916. doi: 10.1371/journal.pone.0288916 (PMC10359008; doi:10.1371/journal.pone.0288916)
Supplement: S4 File — (DOCX) [file pone.0288916.s004.docx]

## S 2: the Interview Guide (English Version)

| **Introduction**  **Opening Statement** | Thank you very much for informing me of your consent to participate in this interview. I would like to ask some questions about the teaching activities that you use or the activities that can be used to improve the academic performance, cognition skills and health-related outcomes of the scholarship exam level students. Any opinion that you would share is highly appreciated, as that will be helpful for our research team to develop an activity-based intervention for school kids. To make sure that I do not miss the data that you provide, would you like me to record our conversation? Of course, this conversation is confidential and, I ensure your privacy. Furthermore, I am using your responses only for the research-related purposes. I will permanently delete all the primary data that you have provided within one year, after completing this study. If you agree, may I ask you to verbally mention that you agree with the interview setting that I have explained to you now? Please feel free to ask any question if you have any at this stage. | |
| --- | --- | --- |
| **Transitioning to...** | **Question(s)** | **Probe(s)** |
| **Warm-up**  Socio-demographic information | First of all, would you like to share something about yourself with me? Including any information about your residency, teaching experience, education level, your age and any achievement of your teaching life... | How about your place of residence?  How many years have you been in teaching now?  Any special experiences that you want to share as a teacher? |
|  | Thank you very much for sharing your information with me. | |
| Approaching towards the interview topic with a convenient talk-related to academic performance | So, please tell me in general, what are expected to teach, how do you teach and how do you evaluate Grade Five students? | Are you always following the teachers’ guide, or do you use your own techniques in addition to the guidelines? |
| **Main body (Topic I to IV)** | Now, I would like to concentrate more on your current teaching practices specifically focusing on mathematics and reading. But, if you like to talk about any other subject in addition to math’s and reading, you are always welcome to speak 😊. | |
| **Topic I**  Academic Outcomes  (Mathematics and Reading) | 1. Please tell me, what are you expected to teach during the mathematics period? Is it just arithmetic or arithmetic and mathematics? | Can you explain to me more on that may be with an example? |
|  | 1. Can you describe the teaching methods that you use to teach mathematics? | Are those methods, formed by you or were you directed to do so by the teacher guides? |
|  | 1. How do you evaluate those students’ mathematics performance? | Do you use standardised tests recommended by some authority? Or do you use your own methods? |
|  | 1. Do you think that the students are needed to be improved in mathematics, if ‘yes’, in what ways? |  |
|  | Let’s talk about the reading performance of the students now. | |
|  | 1. What do you teach your students to improve their reading skills? | For example? |
|  | 1. How do you teach them the reading skills? | Are those methods, formed by you or were you directed to do so by the teacher guides? |
|  | 1. How do you evaluate the reading achievements of the students? | Do you use standardised tests recommended by some authority? Or do you use your own methods? |
|  | 1. Based on your evaluations did you find any need for improvement in reading? | That can be something related to students, or the teaching methods, or the curriculum. Can you tell me more now? |
| **Topic II**  Movement Behaviours and Health Outcomes  (Physical activity levels, Physical fitness/ Aerobic fitness, Stress/ test anxiety) | 1. I would like to bring your attention to the health of students. May I know, how would you like to define ‘health behaviour’ and ‘health’ of a student? |  |
|  | That’s a great definition. Let’s say health behaviour is all about engaging in PA, reducing sedentary behaviour. And health is all about improving physical fitness/ aerobic fitness, living with less exam-related anxiety, and stress. | |
|  | 1. Based on what I defined, could you please tell me whether you are teaching or motivating students to engage in physical activity, and improve physical fitness. 2. could you please tell me whether you are teaching or motivating students to manage stress or cope with test-anxiety. | Can you give me some examples?  Are you required to teach things related to physical activity levels and physical fitness?  What do you do to make them physically active and fit? |
|  | 1. How are you engaging or motivating them to improve mental health-enhancing activities such as coping with test anxiety or stress? | In what ways will you help them to manage stress or cope with test-anxiety?  How about building their confidence to be successful in accomplishing their activities? |
|  | 1. How would you know that they have a good physical and mental health? | How do you evaluate their physical health?  How do you evaluate their mental health?  Is that based on a curriculum guidance or is that based on your own decision-making capacity? |
|  | 1. I would like to get deeper into the mental health aspect a little bit. Do you think that the students are suffering from mental-health problems because of the Scholarship examination? | Can you tell me why do you think so?  May you share some experiences on those aspects with me? |
| **Topic III**  Perceptions on implementing IcPAB | 1. I would like to mention some activities that you can do in the classroom to help you improve the academic performance (more especially reading and mathematics) of the students. I will verbally demonstrate to you an activity now. What is your opinion on that activity? 2. Would you like to share some advice on how and in what ways I should plan those activities? | |
|  | 1. Should you have an opportunity to teach students using physical activities inside the classroom (which would last from five to ten minutes), are you willing to do so? | Can you please, tell me why?  What affected that decision of yours? |
|  | 1. Do you see any challenges in carrying out such activities? And why? |  |
|  | 1. What kind of solutions that we can think of to overcome such challenges? |  |
| **Cool-off** | 1. All right. Thank you very much for sharing your expertise with me. In addition to the questions that I asked, do you have anything else to share with me on this subject? | May be some other concerns of the academic performance, health of students or the teaching methods? |
| **Closure** | This is the end of our interview, then. I will be sent you a top-up fee to your mobile phone number as an appreciation for committing your time during this difficult time. Thank you very much. | |

## S 4: quotes extracted from interviews for data analysis:

| COM-B Components | | Quotes from the interviews |
| --- | --- | --- |
| Capability | Physical capability | *“I am concerned about this… what you are trying to do may not be an easy thing to implement unless the content is physically feasible for teachers who can be relatively old”* [I1, Female, 45 years],  *“There are teachers around their fifties, and they have some physical barriers such as overweight, knee injuries, and fatigue”* [I1, Female, 45 years].  *“… when a teacher gets a little older, it is difficult for them to get up and work. Then they think that it would be better if we focused on more seated work… Now I am forty-seven years old. I took the appointment in 2006. It is not like that I cannot do physical activities with aging. But when you get several fourth or fifth grade classes in a row, you get tired. It can happen.”* [I4, Female, 47 years].  “If you are going to introduce physical activities, think about how they can do those with the masks on... The activities should be designed by considering the social-distancing measures as well. Even though it is hard for us and the students to maintain one meter distance rule, we are always wearing masks.” [I2, Female, 28 years].  *Look, now there are young teachers. There are old teachers. Therefore, it would be good if the physical activities that you recommend us to do in the class are easy for a teacher of all levels to understand and do.* [I3, Female, 37 years]. |
|  | Psychological capability | *“We are trained teachers. We are ready to implement such projects once the required information and training is given”* [I11, Male, 35 years].  “… children are under a lot of pressure. It is not an exam that children willingly face…There are times when the teachers put a lot of pressure on these children. We must cover syllabus… at the same time need to do extra classes prepare them for the scholarship examination… Because of that pressure, students rarely do physical activities inside the classroom… also in the school… Therefore, doing activities in a classroom as you mentioned will be challenging if it takes lots of time from our teaching time…” [I17, Female, 51 years].  “*We at least did practical lessons in the syllabus, past. I am not sure if this answers your question. However, now with that corona, with the problems it had on rules, we didn't do anything for physical health. When there was no such thing, these children were given daily physicals in the morning”.* [I3, Female, 37 years].  *“At grade five, we mostly do is seated work. In any case, the child is not kept seated all day. For a while, those children stand and read or song like that. We do things like that sometimes. Therefore, teachers have that psychological capacity to do activities if instructions are given”* [I3, Female, 37 years].  *“Actually, we have increased the sitting time of the children... we ourselves have increased it... teachers are to be blamed too for that the teachers... … the scholarship exam means a lot to the teachers because they are classified based on the scholarship results of the students. There is an activity in almost every lesson. Now unlike before, every lesson has an activity. Although there is an activity, we do not do the activities. Most of the time the environment is not enough to do that activity in some places. Therefore, I do not know if all teachers can set their minds to do the programme that you suggest”* [I15, Male, 38 years].  *“…because you do too much book work. You do after school classes continuously in the evening too, then that child can't bear it. Because of the pressure that, the child cannot stay focused in the class…get stressed. Consequently, children refuse to go to school because of that. That means the child has too much work, and stressful, right? The child is not emotionally fit to do studies. Teachers know this. Therefore, teachers will be happy to do some physical activities in the class to help the students to forget their fatigue and pressure”* [I16, Female, 50 years]. |
| Opportunity | Physical opportunity | “… you should always think about the amount of space… Because in many schools, after placing 40 or more students in the class, with tables and chairs … the actual space is packed. So, there is very little opportunity to do physical activities in the classroom. Therefore, you should make sure that the activities that you suggest can be done without bumping into the body of others... I mean it would be good if the teacher gets an assessment and confidence that they can do it. Because in the end, if the children go to do the said activities and bump into others, fall to the ground, injure their legs, head, or hands, it is very difficult to deal with those problems in a school” [I11, Male, 35 years].  “… around three hours and twenty minutes per week have been reserved for physical fitness. But there is no way that it is usually done all the time. We use that time sometimes for the morning assembly, to preach the religion... and when it rains or experience bad weather, we cancel outdoor activities... when there are problems... physical active lessons done less. Because we can't take the children outside on a rainy day or a foggy day. Now, especially in a region like ours, it is too foggy in the morning anyway. There are many such problems.” [I2, Female, 28 years].  “Think of being creative to introduce your project. For example, if you tell us that we need more technology, if you tell us to use computers or use something like a television to do activities with children, that kind of thing is not suitable for all schools… many schools in Sri Lanka do not have the resources to try as much as possible. Therefore, it would be good if the activities were introduced to us in such way that we can do them with limited physical resources available in a classroom… even in a rural school with the least resources.” [I7, Female, 36 years].  “For example, I used to work in ... That school was situated in a very difficult location. Children came to school three or four kilometres on foot, jumping over rocks and running through the tea plantations. Such a child does not need any more physical activity. They were also cutting paddy with fathers after school. But in this school, the situation is different… Children, directly get out of the car, right near the school gate… not even walking a kilometre. So that child needs a lot of activity. Then it is not very practical to give the same activity break to a rural child and an urban child. May be some might have special needs or health issues too… it would be better to think of the safety of the kids as well.” [I6, Male, 40 years]. |
|  | Social opportunity | “If a programme like this is recommended at the ministry level, or from the regional office itself... that means if the officials supervise whether this programme will be implemented, teachers are more likely to do it because of the monitoring... if you want to do this work within the expected time, it would be good if we were constantly reminded. Because with the work we have, sometimes we can do those activities for a day or two. But we will go back to our usual routine after that. Therefore, I suggest that either higher-up occasionally come to school and chat with the teachers and give us the support we need, it is a good thing to make the project successful.” [I7, Female, 36 years].  “Anyway, the heavy syllabus should be covered. Two months before the exam, really! there is pressure on the children. Then the children always ask to go to the toilet every ten minutes. Asking to drink water, asking to fill water. Actually, it is not that they want to go to the toilet. They actually want to go out for few minutes. May be if you can use that little minutes to give the students a refreshment that would be great... But please, do not take ten, twenty minutes for an activity...” [I10, Female, 27 years].  *“…it would be better to have a monitoring system to make sure that the activities are done every day… May be not only a record book? Otherwise, I can just mark that I did this today. Only if my heart is honest, I cannot lie. Otherwise, no one knows. Even if I didn't do it, I could mark that I did”* [I5, Male, 38 years].  *“providing clear guidelines about the activities and having an informed decision at least by the principal to use the physical activities breaks in classes will give the teachers a bit of outside pressure. That pressure will motivate the teachers to do the activities that you suggest”* [I15, Male, 38 years] |
| Motivation | Reflective motivation | “… if we have an understanding from some of the things we hear and see, that not only children but anyone who is active can get good physical benefits. However, with the tight syllabus, especially with this scholarship exam, it is difficult to constantly involve those children to do physical activities with the many duties assigned to us... may be if you convince the teachers…if a child is completely active for a certain amount of time a day… then their intelligence will really grow, or confirm that the child will pass the scholarship because of that... I mean, anything about seated time and education or… with acceptable evidence... If there is an understanding at the national level about your programme... it will be a reason for the success of the programme you are trying to bring...” [I18, Male, 35 years]  “When the students are getting closer to the scholarship examination date, they seem really stressed or panicked. If the activities that you are going to introduce will ease their psychological burden and help them to learn well, I personally am motivated to implement those activities. I mean at least the student will be less lethargic and smile often…” [I1, Female, 45 years]  *“It is an obvious thing that the scholarship examination is a stressor for these little ones, right? Maybe you can emphasise more on that and convince the teachers how the activities can help the students to release stress? Because no teacher wants the pupils to become stressed because of this competition created for them in a very little age”* [I19, Female, 53 years]  *“This is what I think… teachers like if any outside programmes can help the students to learn some skills that are crucial to perform well in the scholarship exam. Say it is something to memorise multiplication table. Do you think you can share information with teachers about how your activities can be helpful the students to memorise multiplication table.? The, teachers will motivate to involve in your project”* [I120, Male, 38 years]. |
|  | Automatic motivation | “There was this doctor... When he comes and meets Grade Five teachers in my previous school, he taught us a lot. After I came to this school, I did not meet that doctor. He said…give them the opportunity to draw more pictures, talk to them more, keep them in the front rows and do not blame them if they did not bring a book. I mean we would follow that advice for a week or few days… when time passes, we forget the advice…we become our old selves again. It is neither the teacher's fault nor the child's fault. It is the workload. Therefore, we need regular push with information or reinforcement to motivate ourselves…to help the students to experience happiness when they learn.” [I6, Male, 40 years].  “No problem, I like what you are going to do. Because kids do not like to sit still… need to jump up and shout. That means they are happy. We also will feel a difference, may be for few minutes. Because … if you allocate forty minutes for one subject, if there are forty students in the class, the time that can be given to one child is one minute....” [I9, Male, 35 years].  *“I definitely think that not if your project comes, there can be successful results in the classroom. Nowadays, you should not let the children feel that their learning in the classroom is a boring thing. It is clear that we must have the strategies to create a happy classroom for the child. Therefore, I like to do this activity project”* [I115, Male, 38 years]. |
