# Supplementary material for: Identifying opportunity, capability and motivation of Sri Lankan 5th grade schoolteachers to implement in-classroom physical activity breaks: A qualitative study
Source: PLoS One. 2023 Jul 20;18(7):e0288916. doi: 10.1371/journal.pone.0288916 (PMC10359008; doi:10.1371/journal.pone.0288916)
Supplement: Attachment — Submitted filename: Response to Reviewers.docx [file pone.0288916.s005.docx]

**Dear Dr. Stevo Popovic,**

Thank you very much for inviting us to submit a revised version of the manuscript addressing the points raised by reviewers. We have carefully worked on the revision based on the comments of reviewers. Below are our responses to the reviewers, and we are happy to submit the revised manuscript herewith. Thank you.

# Response to the Editor

Additional Editor Comments (if provided):

I am so pleased to finalize the first review round and request minor revision from the authors. The authors need to revise the manuscript according to the reviewers requests and submit revised manuscript and letter to the reviewers as soon as possible.

*Response: Thank you very much for informing us about the results in the first review round. We are pleased to accept the comments given by the reviewers and revise the manuscript accordingly.*

# Response to Reviewers

1: Is the manuscript technically sound, and do the data support the conclusions?

a
The manuscript must describe a technically sound piece of scientific research with data that supports the conclusions. Experiments must have been conducted rigorously, with appropriate controls, replication, and sample sizes. The conclusions must be drawn appropriately based on the data presented.

Reviewer #1: Yes

Reviewer #2: Yes

*Response: Thank you very much for your positive comments.*

2: Has the statistical analysis been performed appropriately and rigorously?

Reviewer #1: N/A

Reviewer #2: Yes

*Response: Thank you very much for your positive comments.*

3: Have the authors made all data underlying the findings in their manuscript fully available?

The [PLOS Data policy](about:blank#sharing) requires authors to make all data underlying the findings described in their manuscript fully available without restriction, with rare exception (please refer to the Data Availability Statement in the manuscript PDF file). The data should be provided as part of the manuscript or its supporting information, or deposited to a public repository. For example, in addition to summary statistics, the data points behind means, medians and variance measures should be available. If there are restrictions on publicly sharing data—e.g. participant privacy or use of data from a third party—those must be specified.

Reviewer #1: Yes

Reviewer #2: Yes

*Response: Thank you very much for your positive comments.*

4: Is the manuscript presented in an intelligible fashion and written in standard English?

PLOS ONE does not copyedit accepted manuscripts, so the language in submitted articles must be clear, correct, and unambiguous. Any typographical or grammatical errors should be corrected at revision, so please note any specific errors here.

Reviewer #1: Yes

Reviewer #2: Yes

*Response 1: Thank you very much for your positive comments.*

5: Review Comments to the Author

Reviewer #1: It is necessary to precisely define the sample of respondents in the abstract. Number, age, gender...
I like the idea of this study. Based on this idea, it will be possible to construct policies to improve physical activity and to construct tools for assessing and controlling physical activity.
I think it will contribute to the development of science and practice in the future.

*Response: We thank you for your positive comments and suggestion. We have added sample characteristics information to the abstract. It is presented as follows:*

***“****21 out of the recruited teachers responded to the full study. The mean age of respondents was 39.24 years old ranging from 27 years to 53 years. Teaching experience of the respondents ranged from three to 37 years, and 57% were female. Three teachers had a degree with a teacher training diploma, while others were having General Certificate of Education in Advanced Level with a teacher training diploma as the highest education qualification.****”***

Reviewer #2: The topic of the manuscript is excellent and current. The manuscript is very well methodologically written. The introduction is excellently conceived, in accordance with the objective of the research. The methodology is very well applied, the results are in accordance with the data obtained by statistical analysis. Discussion and Conclusion in accordance with the obtained results, with the presented limitations of the study and recommendations.

*Response: Thank you very much for your positive comments.*

6: PLOS authors have the option to publish the peer review history of their article ([what does this mean?](about:blank#loc-peer-review-history)). If published, this will include your full peer review and any attached files.

If you choose “no”, your identity will remain anonymous but your review may still be made public.

**Do you want your identity to be public for this peer review?** For information about this choice, including consent withdrawal, please see our [Privacy Policy](about:blank).

Reviewer #1: **Yes:** Bojan Masanovic

Reviewer #2: **Yes:** Jovan Gardasevic, University of Montenegro

*Response: Thank you for agreeing to make available your information in public. We are honoured to obtain your review of our paper.*

In the case of questions, please do not hesitate to contact me. We are looking forward to your reply.

Sincerely yours

**Dr Duan Yanping**

On behalf of all co-authors

Hong Kong Baptist University

Hong Kong

28^th^ April 2023
